# Integrating science to address food and health within Global Agenda 2030

**DOI:** 10.1038/s41538-021-00088-1

**Published:** 2021-04-07

**Authors:** Gordon A. McBean

**Affiliations:** Department of Geography and Environment and Institute for Catastrophic Loss Reduction, Western University, London, ON Canada

**Keywords:** Agriculture, Business and industry

## Abstract

When recovering from the pandemic, it is important for Planet Earth to address the Global Agenda 2030, including food and water crises, and to bounce forward sustainably. The World Economic Forum’s Global Risk Report and Global Agenda 2030 provide a framework for action and an integrated global science agenda response, involving food and health, is essential. The UN 2021 Summit on Food Systems provides an opportunity for the global science community to come together to address the Summit’s Action Tracks, including building resilience to vulnerabilities, shocks and stresses. There would be major global benefits to have an international scientific network working with the UN to address the mandates of the UN Food Summit and Global Agenda 2030.

## Global Agenda 2030, food and the pandemic world

In 2015, the Sustainable Development Goals^1^, the Paris Agreement^2^ of the UN Framework Convention on Climate Change, and the Sendai Framework on Disaster Risk Reduction 2015–2030^3^ were created and endorsed by almost all countries. The New Urban Agenda^4^ was agreed to in 2016. In addressing this integrated Global Agenda 2030^5^ with its complexities and challenges, it is important that the global science community comes together.

Sustainable development^6^, defined as: “*Humanity has the ability to make development sustainable - to ensure that it meets the needs of the present without compromising the ability of future generations to meet their own needs*”, links social, economic, technology, science and environmental issues and calls on the science and technology communities to enhance the ability to meet present and future needs together. There are 17 Sustainable Development Goals with 169 targets. These Goals link with the Paris Agreement (Goal 13—climate action), the Sendai Framework (Goal 9—resilient infrastructure; Goal 11—safe and resilient communities) and other components of the Global Agenda.

Food is key across many Goals, with Goal 2 (zero hunger) being to: “*End hunger, achieve food security and improved nutrition and promote sustainable agriculture*.” It is strongly connected with Goals 3—health, 1—poverty, 13—climate, 14—life below water, 15—life on land, 6—clean water and sanitation and 9—industry, innovation and infrastructure. Sustainable development and livestock policy is one example of connections^7^. All Goals interact^8^ and understanding their interactions and bringing science together to address them is key to unlocking their full potential and ensuring that progress made in some areas is not made at the expense of progress in others. Goals 16-good governance and 17-means of implementation are keys to turning the potential for synergies into reality.

The 2015 Paris Agreement strengthens the global response to the threat of climate change, in the context of sustainable development and efforts to eradicate poverty, through mitigation-emissions reductions, including the food sector (production and processing of food), and adaptation to the adverse impacts of climate change, to foster climate resilience and to gain the benefits of a warming climate, where they exist^9^. The UN Secretary-General António Guterres, at the 2019 Climate Action Summit, stated^10^: “*Climate change is the defining challenge of our time”* and emphasized the need to address the risks^11^.

The Sendai Framework for Disaster Risk Reduction 2015–2030 links with development, climate change and other aspects including building resilience and achieving the global goal of eradicating poverty. Its priorities are: improved understanding; strengthening governance; investing for resilience; and enhancing disaster preparedness and to “*Build Back Better*”. The 7 global targets include to substantially reduce, by 2030, disaster mortality, numbers of affected people, economic loss, damage to critical infrastructure and disruption of basic services and to provide access to multi-hazard early warning systems and disaster risk information and assessments. The New Urban Agenda sets a new global standard for sustainable urban development to plan, manage and live in cities. The Agenda provides guidance for achieving the Sustainable Development Goals and provides the underpinning for actions to address climate change.

For Planet Earth, the Covid-19 pandemic has already caused over 1 million deaths and the impacts are quite varied^12^ and it is a priority for action. However, recovery from the pandemic provides an opportunity for nations to work together to do this most effectively while also addressing Global Agenda 2030, the issues of food, nutrition and health, including the safety and integrity of the food supply^13^. The 2019 International Forum on Food Safety and Health^14^ and the 2nd International Union of Food Sciences and Technology (IUFoST) Global Food Summit - Strategies for a Sustainable and Resilient Food System^15^ discussed the opportunities and challenges for integrated science-based strategies and their implementation to achieve Global Agenda 2030 in recovering from Covid-19 and in bouncing forward to sustainability.

The IUFoST Roundtable on COVID-19 and Food Safety^16^ in 2020 addressed major reviews of food systems with special emphasis on resilience. Safe food practices should be reinforced by promoting good food safety habits developed during the pandemic. Food scientists and technologists and entrepreneurs^17^ should have a strong role in government policy and contingency planning to ensure the resilience of the food supply chain in responding to future pandemics, recognizing the need to “*enhance food systems resilience to shocks like this and ongoing stresses like climate change*”^18^.

## Global Risk Reports

The World Economic Forum (WEF)^19^ prepares annual Global Risk Reports which rank the global risks (economic, environmental, geopolitical, societal, technological) by impacts and likelihood. A global risk is an uncertain event or condition that, if it occurs, can cause significant negative impacts for several countries or industries within the next 10 years. The 2020^20^ Report was published in January 2020 and notes that: “*For the first time in the history of the survey, climate-related issues dominated all of the top-five long-term risks by likelihood*”. Extreme weather events (storms, floods, …) “*causing major property, infrastructure, and/or environmental damage as well as loss of human life*”, are ranked as the highest in likelihood and 4th highest in terms in impacts. Climate Action Failure (“*The failure of governments and businesses to enforce or enact effective measures to mitigate climate change, protect populations and help businesses impacted by climate change to adapt*”) ranked as the number one risk by impact and the number two by likelihood. Clearly these two risks are highly linked.

Biodiversity loss (“*Irreversible consequences for the environment, resulting in severely depleted resources for humankind as well as industries*”) is ranked as the 3rd highest risk in terms of impact and 4th highest in terms of likelihood. Water Crises (“*significant decline in the available quality and quantity of fresh water resulting in harmful effects on human health and/or economic activity*”) are ranked as the 5th highest in impact and 8th highest in likelihood.

Food crises (“*inadequate, unaffordable, or unreliable access to appropriate quantities and quality of food and nutrition on a major scale*”) are not ranked in top 10 but the report examines the intersection of food systems with other risks, such as biodiversity and climate. In their analysis of food insecurity, the Report notes that biodiversity underpins the world’s food system^21^ and how it creates and maintains healthy soils, pollinates plants, purifies water and protects against extreme weather events^22^. The Intergovernmental Panel on Climate Change’s (IPCC) Special Report on Climate Change and Land^23^ addresses the interconnections between climate change, desertification, land degradation, sustainable land management, food security and greenhouse gas fluxes in terrestrial ecosystems.

The Global Risk Report also assesses the interconnections between issues. Based on the experts’ assessments, there are clearly major connections between climate action failure, extreme weather events and biodiversity loss and these link with water crises. Food crises interconnect with environmental risks and water crises, as well as the axes of risks of governance failure, highlighting their importance at global, national and urban levels.

Climate change and the current COVID-19 pandemic both demonstrate the importance of enhancing resilience as key for disaster risk reduction. It is very important that hazard and risk information and strong science-policy-society interfaces result in better risk-informed public and private decision-making and investment for long-term resilience. It is essential that there be an all-hazards approach to achieve risk reduction for sustainable development and climate change adaptation^24^. Poverty, rapid urbanization, weak governance, the decline of ecosystems and climate change are driving disaster risks around the world. The Paris Agreement and the Sendai Framework provide strategic actions for achieving the Sustainable Development Goals.

## Global science programmes addressing Global Agenda 2030

The World Climate Research Programme (WCRP)^25^ was established in 1980 to address: (1) “*to what extent can the global climate be predicted*”; and (2) “*how have humans influenced the climate?'*” The scientific outputs from WCRP have been the prime basis for the assessments of the Intergovernmental Panel on Climate Change and guiding the Climate Convention. The Integrated Research on Disaster Risk (IRDR) Programme^26^, created in 2008, has the objectives of (1) characterization of hazards, vulnerability and risk; (2) understanding decision-making in complex and changing risk contexts; and (3) reducing risk and curbing losses through knowledge-based actions. The risk interpretation to actions by individuals, governments and private sector in the context of disaster risk reduction is an important issue. The IRDR provides scientific input to the Sendai Framework.

The Future Earth Programme: Research; Innovation; Sustainability^27^ brings science together across disciplines and the issues of sustainable development. Future Earth plays a significant role at the interface between science and international policy through the SDGs and Paris Agreement, and partners with IRDR, with respect to the Sendai Framework. With 19 Global Research Projects^28^, Future Earth has also developed Knowledge-Action Networks^29^ (KANs) which are collaborative frameworks that facilitate highly integrative sustainability research to inform solutions for complex societal issues. One KAN is on Emergent Risks and Extreme Events. The Health and Wellbeing in the Changing Urban Environment^30^ programme focuses on systems analyses approaches towards understanding health and wellbeing in urban settings.

At the World Economic Forum in January 2019, the UN Secretary-General^31^, referring to the growing risks in a shrinking world, stated: “*If I had to select one sentence to describe the state of the world, I would say we are in a world in which global challenges are more and more integrated, and the responses are more and more fragmented, and if this is not reversed, it’s a recipe for disaster.”* There is need for an integrated approach to addressing hazards, including Food Crises and Infectious Diseases, and for integrated, strategic scientific-based actions to reduce risks and impacts to meet the global societal needs.

## Bouncing forward sustainably post COVID-19

The International Institute for Applied Systems Analysis (IIASA)^32^ and the International Science Council (ISC)^33^, global science organizations with broad memberships across issues, have created the Consultative Science Platform^34^: Bouncing Forward Sustainably Post COVID-19 to bring together global experts to define and design sustainability pathways that will enable building-back a more sustainable post COVID-19 world. Their four themes are Governance for Sustainability, Strengthening Science Systems, Sustainable Energy, and Resilient Food Systems. In addressing the issues of governance for sustainability, a systemic and compound risk perspective for addressing risks is recommended and risk governance is fundamental for achieving sustainability amid multiple crises and uncertain events^35^. With systemic risks, failure in one sub-unit or part of the system can lead to triggering cascading events in other system units leading to major disturbances or even complete failure of the whole system. It is recommended that, at national-local governance levels, it is important to put systemic resilience at center-stage and maintain transparency and accountability. Increased awareness of compound and systemic risks would benefit multi-level governance and a joint vision of a more sustainable, resilient post-pandemic economy and society is important^36^.

## Addressing food science and technology in the Global Agenda

The 2010 Cape Town Declaration of the 15th World Congress of Food Science and Technology^37^ states: *“… need for ongoing active collaboration and exchange of information with other bodies, … particularly those of the sciences contributing to or related to the multi-disciplinary subject which is food science*”. Global food systems are critical for the sustained future of Planet Earth and transformations of the food system are essential^38^. SDG 2 (zero hunger) Target 2.4 is to, by 2030, “*Ensure sustainable food production systems and implement resilient agricultural practices that increase productivity and production, that help maintain ecosystems, that strengthen capacity for adaptation to climate change, extreme weather, drought, flooding and other disasters and that progressively improve land and soil quality*”. It has been shown^39^ that the scientific targets for healthy diets from sustainable food systems are intertwined with all the SDGs and it is required that the scientific targets be adopted by all sectors to stimulate a range of actions from individuals and organisations working in all sectors and at all scales. Many SDGs are critically reliant on the food chain and “*the missions involve new greater interdisciplinary collaboration and require development of new measurement science*”^40^. The IIASA – ISC Consultative Science Platform on Resilient Food Systems^41^, that focussed on building resilient food systems in the recovery process, noted that it is important to: “*(1) reorient food system architecture towards an emphasis on resilience and equity; (2) make human and planetary health concerns an integral component of food systems; (3) secure innovation, technology diffusion and upscaling of sustainable practices; (4) strengthen collaboration and partnerships for transformative actions; and (5) reform the science-policy interface for strategic decision making.*” In food systems, it is important to recognize the importance of the different sectors, including the multiple issues in food crop production, the processing sector and the transportation and distribution and consumption sectors. There are opportunities for augmenting ecosystems for food production^42^. With a growing global population, feeding the population with diminishing natural resources, whilst ensuring the health of people and the planet, it important to assess the roles of agricultural and food science in meeting the global food demand^43^. In addition to the challenges to food and agriculture systems in the face of climate change and global megatrends that are shaping the future world, it is also very important to address the important issues in food processing, which can use food technology to reduce waste and provide a leading role in the overall food supply.

Communicating scientific information so that individuals, private sector or government agencies most effectively respond is important and the communication of the safety of food supply and risks and food information and consumer engagement^44^ are important. Promoting public science literacy is also important (Beijing Declaration for Promoting Public Science Literacy Across the World)^45^ and science communities need to more effectively communicate in an effective and coordinated manner.

## UN 2021 Summit on Food Systems

In 2021, UN Secretary-General Guterres will convene a Food Systems Summit^46^ as part of the Decade of Action to achieve the Sustainable Development Goals (SDGs) by 2030. The Summit’s plan is to initiate actions to deliver progress on all 17 SDGs, noting “*each of which relies to some degree on healthier, more sustainable and equitable food systems*”. The Summit will address across the issues in producing, processing, transporting and consuming food. It is noted that “*too many of the world’s food systems are fragile, unexamined and vulnerable to collapse, as millions of people around the globe have experienced first-hand during the COVID-19 crisis”* and failure has major broad impacts. The mandate of the UN Food Summit is to deliver the following outcomes, summarized as: “g*enerate significant action and measurable progress towards the 2030 Agenda for Sustainable Development; raise awareness and elevate public discussion about how reforming our food systems can help us all; develop principles to guide governments and other stakeholders looking to leverage their food systems to support the SDGs (which food systems play a central role in building a fairer, more sustainable world);* and *creation of a system of follow-up and review to ensure that the Summit’s outcomes continue to drive new actions and progress*”.

In alignment with the Summit’s objectives, five Action Tracks^47^ are proposed for (1) ensuring access to safe and nutritious food for all, (2) shifting to sustainable consumption patterns, (3) boosting nature-positive production at scale, (4) advancing equitable livelihoods and (5) building resilience to vulnerabilities, shocks and stresses. The Action Tracks are recognized as not being separate, with possible trade-offs between tracks that need to be addressed and to identify solutions that can deliver wide-reaching benefits. The Summit recognizes the need to draw on the expertise of actors from across the world’s food systems and to bring together key players from the worlds of science, business, policy, healthcare and academia, as well as farmers, indigenous people, youth organizations, consumer groups, environmental activists, and other key stakeholders.

## Concluding remarks

The 2021 Summit on Food Systems provides an opportunity for the global science community to respond and work together to address the food and health issues within the Global Agenda 2030 and the need to provide safe, secure (adequate, affordable and accessible) and sustainable (environmental and economic) food. These grand challenges highlight the needs for transformative research^48^ and integrative science, working across disciplines and fields with joint, reciprocal framing, design, execution and application of research; working globally; and with society^49^. It is important to engage decision makers, policy shapers, practitioners, as well as actors from civil society and the private sector as partners in the co-design and co-production of solutions-oriented knowledge, policy and practice. Integrated science for transformative research is needed to address the Global Agenda 2030. There is need for more effective science for policy translation, including integrated assessments of food, health and water crises and climate change, with a growing and evolving human sector on the Planet to effectively recover and bounce forward from Covid-19.

In responding to the 2021 Food Summit opportunity, the IUFoST^50^, International Institute for Applied Systems Analysis (IIASA) and the International Science Council (of which IUFoST is a member) and their broad scientific communities could address the Summit’s Action Tracks, including building resilience to vulnerabilities, shocks and stresses. Food science and technology roles in building safe, secure and sustainable food systems are important as having food supply chains that are more resilient would have major societal benefits. The International Institute for Applied Systems Analysis (IIASA) and the International Science Council could extend their Consultative Science Platform: Bouncing Forward Sustainably Post COVID-19 with a co-focus on governance for sustainability and resilient food systems, bringing together global science programmes and other appropriate organizations. There would be major global benefits for the Global Agenda to have an international scientific network working with the UN to address the mandates of the UN Food Summit and Global Agenda 2030.
